# Cost-effectiveness of voluntary medical male circumcision for HIV prevention across sub-Saharan Africa: results from five independent models

**DOI:** 10.1016/S2214-109X(22)00515-0

**Published:** 2022-12-20

**Authors:** Loveleen Bansi-Matharu, Edinah Mudimu, Rowan Martin-Hughes, Matt Hamilton, Leigh Johnson, Debra ten Brink, John Stover, Gesine Meyer-Rath, Sherrie L Kelly, Lise Jamieson, Valentina Cambiano, Andreas Jahn, Frances M Cowan, Collin Mangenah, Webster Mavhu, Thato Chidarikire, Carlos Toledo, Paul Revill, Maaya Sundaram, Karin Hatzold, Aisha Yansaneh, Tsitsi Apollo, Thoko Kalua, Owen Mugurungi, Valerian Kiggundu, Shufang Zhang, Rose Nyirenda, Andrew Phillips, Katharine Kripke, Anna Bershteyn

**Affiliations:** a Institute for Global Health, UCL, London, UK; b Department of Decision Sciences, University of South Africa, Pretoria, South Africa; c Burnet Institute, Melbourne, VIC, Australia; d Avenir Health, Takoma Park, MD, USA; e Avenir Health, Glastonbury, CT, USA; f Health Economics and Epidemiology Research Office, Department of Internal Medicine, School of Clinical Medicine, Faculty of Health Sciences, University of the Witwatersrand, Johannesburg, South Africa; g Department of Global Health, Boston University School of Public Health, Boston, MA, USA; h Centre for Infectious Disease Epidemiology and Research, University of Cape Town, Cape Town, South Africa; i Ministry of Health, Lilongwe, Malawi; j International Training and Education Center for Health, Department of Global Health, University of Washington, Seattle, WA, USA; k Center for Sexual Health and HIV/AIDS Research, Harare, Zimbabwe; l Department of International Public Health, Liverpool School of Tropical Medicine, Liverpool, UK; m National Department of Health, Pretoria, South Africa; n Division of Global HIV/AIDS and Tuberculosis, Centers for Disease Control and Prevention, Atlanta, GA, USA; o Centre for Health Economics, University of York, York, UK; p Global Development Program, Bill & Melinda Gates Foundation, Seattle, WA, USA; q Population Services International, Washington, DC, USA; r United States Agency for International Development, Washington, DC, USA; s Department of HIV and AIDS, Ministry of Health Malawi, Lilongwe, Malawi; t Institute of Social and Preventive Medicine, University of Bern, Bern, Switzerland; u Ministry of Health and Child Welfare, Harare, Zimbabwe; v The Global Fund to Fight AIDS, Tuberculosis, and Malaria, Geneva, Switzerland; w Department of Population Health, New York University Grossman School of Medicine, New York, NY, USA

## Abstract

**Background:**

Voluntary medical male circumcision (VMMC) has been a recommended HIV prevention strategy in sub-Saharan Africa since 2007, particularly in countries with high HIV prevalence. However, given the scale-up of antiretroviral therapy programmes, it is not clear whether VMMC still represents a cost-effective use of scarce HIV programme resources.

**Methods:**

Using five existing well described HIV mathematical models, we compared continuation of VMMC for 5 years in men aged 15 years and older to no further VMMC in South Africa, Malawi, and Zimbabwe and across a range of setting scenarios in sub-Saharan Africa. Outputs were based on a 50-year time horizon, VMMC cost was assumed to be US$90, and a cost-effectiveness threshold of US$500 was used.

**Findings:**

In South Africa and Malawi, the continuation of VMMC for 5 years resulted in cost savings and health benefits (infections and disability-adjusted life-years averted) according to all models. Of the two models modelling Zimbabwe, the continuation of VMMC for 5 years resulted in cost savings and health benefits by one model but was not as cost-effective according to the other model. Continuation of VMMC was cost-effective in 68% of setting scenarios across sub-Saharan Africa. VMMC was more likely to be cost-effective in modelled settings with higher HIV incidence; VMMC was cost-effective in 62% of settings with HIV incidence of less than 0·1 per 100 person-years in men aged 15–49 years, increasing to 95% with HIV incidence greater than 1·0 per 100 person-years.

**Interpretation:**

VMMC remains a cost-effective, often cost-saving, prevention intervention in sub-Saharan Africa for at least the next 5 years.

**Funding:**

Bill & Melinda Gates Foundation for the HIV Modelling Consortium.

## Introduction

Voluntary medical male circumcision (VMMC) has been a globally recommended HIV prevention strategy since 2007, particularly in countries of sub-Saharan Africa with low coverage of male circumcision and high HIV prevalence.^1^ Circumcision has been reported to reduce the risk of HIV acquisition by around 60%^2^, ^3^ and VMMC has been scaled up in 15 eastern and southern African countries since 2011.^4^

The vast majority of VMMCs performed in Africa are supported by the US President's Emergency Plan for AIDS Relief (PEPFAR), with more than 25 million VMMCs by 2020 (representing >80% of total VMMCs) in the 15 countries mentioned.^1^, ^4^, ^5^ Furthermore, in evaluations to date, VMMC is cost-effective and even cost-saving in a range of settings, including Malawi and South Africa.^6^, ^7^, ^8^, ^9^, ^10^, ^11^, ^12^, ^13^, ^14^

At the same time, the scale-up of antiretroviral therapy (ART) programmes and prevention interventions such as pre-exposure prophylaxis has resulted in a decline in HIV incidence in several countries. Hence, it is unclear whether VMMC programmes are still cost-effective. Consequently, it is valuable to reassess under what conditions, particularly according to the current HIV incidence, the provision of VMMC services is likely to continue to represent a cost-effective use of scarce HIV programme resources.

Using five existing and well described HIV mathematical models, we compared the effect of two policy options: (1) continuation of VMMC for the next 5 years in men aged 15 years and older; and (2) no further VMMC in any age group in regions across sub-Saharan Africa, with particular focus on Malawi, South Africa, and Zimbabwe. We analysed model outputs over 50 years to capture the full effects of VMMC for the two policy options, including effect on HIV incidence, mortality, and cost-effectiveness.

**Research in context**

**Evidence before this study**

Since January, 2007, voluntary medical male circumcision (VMMC) has been a globally recommended HIV prevention strategy, particularly in areas with high HIV prevalence. On June 15, 2020, we searched the Web of Science using the search terms (“VMMC” or “voluntary medical male circumcision” or “circumcision”) AND (list of countries in sub-Saharan Africa) AND (cost-effective*) to identify all articles assessing the cost-effectiveness of VMMC. No date restrictions were applied to the search. Since 2011, VMMC has been scaled up in 15 countries in sub-Saharan Africa and has been shown to be cost-effective by several modelling groups. However, since then, antiretroviral therapy programmes have also been scaled up. Hence, it is not known whether the provision of VMMC services continues to represent a cost-effective use of limited HIV programme resources.

**Added value of this study**

This study was a joint modelling analysis using five independent, well established models to compare the continuation of VMMC with no further VMMC, focusing on South Africa, Malawi, and Zimbabwe. We concluded from this study that VMMC continues to represent a cost-effective use of HIV programme resources.

**Implications of all the available evidence**

Despite the scale-up of antiretroviral therapy and low HIV incidence in some settings, the continuation of VMMC for at least the next 5 years is cost-effective in almost all settings considered in this study.

## Methods

### Study design

We compared results from five models—Optima HIV, EMOD, Thembisa, Goals-ASM, and HIV Synthesis—that have been previously described.^15^, ^16^, ^17^, ^18^, ^19^ Key model features are summarised in table 1. VMMC was examined in three sub-Saharan African countries: Malawi, South Africa, and Zimbabwe, and (for the HIV Synthesis model) in a range of setting scenarios intended to represent the breadth of settings within sub-Saharan Africa. These countries and settings represent different HIV epidemics and differences in circumcision prevalence (table 2). VMMC is assumed to reduce female-to-male HIV transmission risk by 58–60% in all models. Non-medical male circumcision, including traditional circumcision, is also assumed to reduce female-to-male HIV transmission risk by the same amount. The models all capture HIV infection transmission dynamics, so the future reduction in transmissions to other sexual partners from men who would otherwise have become infected with HIV are also incorporated. The models furthermore take into account population growth and associated demographic shifts in the countries over time.

**Table 1:** Characteristics of the contributing models

	**Goals-ASM (South Africa, Malawi, Zimbabwe [and 9 others])**	**Optima HIV (South Africa, Malawi, and Zimbabwe)**	**HIV Synthesis (range of setting scenarios)**	**EMOD (South Africa)**	**Thembisa (South Africa)**
Structure	Compartmental model with disaggregation by sex and risk group; 1-year time step	Compartmental model with population disaggregation depending on setting, including sex, age, and risk group; 2·4-month time step	Individual-based stochastic model; 3-month time step	Individual-based stochastic model; daily time step	Compartmental model with disaggregation by sex, age, risk group, and marital status; 1-month time step
Approach to calibration to data and estimates for specific settings	Epidemiological parameters (probability of transmission per act, variation by stage of infection, presence of other STIs, effectiveness of condoms, and ART) are varied to fit the model to prevalence estimates from surveillance and surveys	Epidemiological parameters (probability of transmission per act, variation by stage of infection [informed by CD4 counts and viral load monitoring], HIV testing rate, mortality rate, presence of other ulcerative STIs or tuberculosis, or both, as well as effectiveness of condoms, circumcision, and suppressive or unsuppressive ART) are varied to fit the model to prevalence estimates from surveillance and surveys	Parameters relating to population characteristics, sexual behaviour (condomless sex) and age–gender mixing, HIV acquisition, HIV testing, natural history (CD4 count and viral load), ART, and risk of AIDS and death are varied within plausible bounds to create a range of setting scenarios; no calibration to a specific country was performed for this exercise	The model is parameterised with epidemiological data including population size, fertility, mortality, voluntary male circumcision coverage, and health-seeking and sexual behaviour; South Africa data on age and sex-specific HIV prevalence, ART coverage, and HIV incidence were used to calibrate the model; calibration was performed using a PSPO algorithm; roulette resampling in proportion to the likelihood of each simulation was used to select 250 model parameter sets	HIV transmission rates per sex act, HIV survival parameters, sexual behaviour parameters, HIV testing rates, and ART initiation rates are varied in calibrating the model to South African HIV prevalence data (antenatal, household, and key population surveys), mortality data, HIV testing history data, ART programme data, and paediatric HIV data, using a Bayesian approach
Sexual behaviour	Behaviours (number of partners, acts per partner, condom use, and needle sharing) differ by risk group: FSW, male clients, men and women with non-regular partners, faithful couples, MSM, and PWID	Behaviours (type of sexual partners [regular, casual, or commercial]; injecting), acts per partner, condom use, and needle sharing differ by age and risk group (FSW, clients of sex workers, MSM, and PWID)	Two types of condomless sexual partnerships (long-term and short-term); long-term partnerships are remembered over time; rates of condomless sex contact depends on age, type of partnership, and sex; MSM and PWID are not explicitly modelled	Four types of sexual partnerships (marital, informal, transitory, and commercial) are remembered over time and formed according to specifiable partner age patterns	Three types of sexual partnership (marital or cohabiting, short-term, and commercial), with different coital frequencies and condom use for each; rates of short-term and commercial sex contact depend on risk group (high or low), age, marital status, and sex; separate assumptions for MSM
HIV acquisition determinants (including prevention interventions)	Acquisition risk depends on characteristics of the individual (number of partners, circumcision status [men], and PrEP use), the partner population (HIV prevalence, ART use, and stage of infection), and the partnerships (acts per partner, prevalence of other STIs, type of act, and condom use)	Acquisition risk depends on characteristics of the individual (number of partners, and number of injections), and the partnerships (type of partner, acts per partner, condom use, circumcision status [men], MTCT status, PrEP and PEP use, and receptive needle sharing), and HIV status by population (HIV testing, HIV diagnosis, HIV prevalence, suppressive or unsuppressive ART use, and stage of infection)	Acquisition risk depends on characteristics of the individual (age, sex, number of condomless sex partners, circumcision status [men], and PrEP use), the partner population (HIV prevalence, and viral load) and the partnership (type of condomless sex partnership); transmission of virus with specific drug resistance mutations is modelled	Acquisition risk depends on characteristics of the individual (number of partners, circumcision status [men], condom use, other STIs, and acts per partner) and of the partner (HIV prevalence, ART use, and stage of infection)	Acquisition risk per sex act depends characteristics of the individual (age, sex, risk group, type of relationship, circumcision status [men], and PrEP use) and HIV positive partner characteristics (condom use, acute infection, CD4 count, ART status, and viral suppression)
HIV natural history	Rate of decline of CD4 count off ART depends on current CD4 count and age; mortality off ART depends on sex, age, and CD4 count; mortality on ART depends on CD4 count at initiation, age, sex, and duration on ART	Rate of decline of CD4 count and viral load of ART depends on current CD4 count and viral load; change in CD4 count on ART depends on current CD4 count and viral suppression from treatment; mortality both on and off ART depends on current CD4 count and ART status (suppressive or unsuppressive)	Rate of decline of CD4 count dependent on current viral load; viral load increases over time; risk of AIDS and death dependent on current CD4 count, current viral load, age, and current use of co-trimoxazole	HIV prognosis is calculated using a Weibull distribution where the parameters of the distribution are derived from CD4 count and age at the time of infection; CD4 count decreases from the time of infection while off ART; CD4 count increases if an individual enrols for ART; new prognosis is calculated for ART dropouts	After acute infection phase, individual progresses through four CD4 stages in absence of ART, with rates of CD4 progression depending on age and sex; untreated mortality depends on age, sex, and CD4 count
HIV testing and diagnosis	Testing is by modality and population group and determines knowledge of status, but this is not linked to transmission since ART coverage is direct input	Testing is modelled by modality, population group, and year, which determines knowledge of status and allows linkage to care and initiation on ART based on treatment coverage level	Whether a person is tested or not is defined in each period; testing is indicated in antenatal clinic, for symptoms potentially of HIV, and potentially every 6 months for FSW; general testing with various degrees of targeting at people with higher probability of infection and dependent on testing history	HIV testing and diagnosis occurs voluntarily, at antenatal visits, or once symptomatic	Three types of testing in adults: antenatal, testing in patients with opportunistic infections, and general testing; general testing rates vary by age, sex, and testing history
ART	Risk of mortality while on ART is determined by age, sex, CD4 count at treatment initiation, and duration on treatment	Risk of mortality while on ART is determined by dynamically changing CD4 counts over the course of treatment	Specific drugs and their current level of activity given drug resistance, and current ART adherence determine viral load, CD4 count change, and risk of further resistance; being currently on ART has an independent effect on risk of AIDS and HIV death over and above these	Risk of mortality while on ART is determined by age, sex, CD4 count at treatment initiation, and duration on treatment	Risk of mortality on ART depends on baseline CD4 count, ART duration, age, and sex
ART interruption	Immediate return to CD4 count at time of treatment initiation; survival progression identical to those who are treatment naive	CD4 count decreases following dropout from ART; prognosis is recalculated on the basis of age and CD4 count at the time of ART dropout; computation of ART prognosis after re-enrolment on treatment is the same as the initial enrolment with prognosis parameters corresponding to age and CD4 count at re-enrolment	Immediate (within 3 months) viral load return to pre-ART concentration, substantial initial decline in CD4 count towards pre-ART nadir	CD4 count decreases following dropout from ART; prognosis is recalculated based on the age and CD4 count at the time of dropping off ART; computation of ART prognosis after re-enrolling is identical to the initial enrolment with prognosis parameters corresponding to the age and CD4 count at re-enrolment	Immediate return of CD4 and viral load to concentrations at time of ART initiation, after an ART interruption; however, no effect of ART interruption on mortality is modelled
MTCT and its prevention	Depends on CD4 count of the mother if no prophylaxis, or prophylaxis regimen; retention on prophylaxis at delivery, duration of breastfeeding, and dropout of prophylaxis during breastfeeding	Dependent on CD4 count of the mother, MTCT coverage, and breastfeeding duration and the percentage of HIV-positive women who breastfeed	Dependent on viral load in the mother at birth	Depends on CD4 count of the mother if no prophylaxis, or prophylaxis regimen; retention on prophylaxis at delivery	Depends on mother's CD4 or acute infection stage (if untreated), duration of ART and type or duration of breastfeeding
Future ART coverage, default	ART coverage (men and women) remains at 2020 levels	Proportion of people on ART including initiation and retention maintained at 2020 levels	Rates of HIV testing, engagement with care upon diagnosis, ART initiation in those engaged, and ART retention remain at 2020 levels	Rates of ART initiation and ART retention remain at 2020 levels	Rates of ART initiation and interruption after 2019 are assumed to remain constant, based on average rates observed over 2014–19
Lower future ART coverage	Linear reduction of 20% in ART coverage (by age and sex) from 2020 to 2040	Proportion of people diagnosed with HIV who have access to ART gradually linearly decreases from 2021 to an overall 20% reduction in ART coverage by 2040	Change in above rates resulting in an overall 20% reduction of ART coverage by 2040	A gradual decrease in ART retention rate resulting in an overall 20% reduction of ART coverage by 2040	Rates of ART interruption are assumed to increase, such that ART coverage by 2040 is 20% lower than in the main scenario
Circumcision	Represented as male circumcision prevalence; does not distinguish between traditional and medical circumcision; 60% lower risk of HIV acquisition	Two types of circumcision were included within the percentage of men ever circumcised: background or traditional male circumcision and circumcision in response to VMMC campaigns; 58% lower risk of HIV acquisition	Two types of circumcision were included within the percentage of males ever circumcised: background or traditional male circumcision and circumcision in response to VMMC campaigns; probabilities of VMMC depend on age and calendar year to reflect span of VMMC prevalence across the region; 60% lower risk of HIV acquisition	Both background or traditional male circumcision and response to VMMC campaigns are considered; circumcision rates depend on age; 60% lower risk of HIV acquisition	Two types of circumcision: background male circumcision (which includes traditional circumcision) and circumcision in response to VMMC campaigns; both rates depend on age; 60% lower risk of HIV acquisition
Calculation of DALYs	Years lost to disability, plus years of life lost, discounted at 3%; years of life lost are incurred in the year of death based on the average life expectancy at the time of death according to a standard life table with life expectancy at birth of 80 years	Years lost to disability plus years life of lost (assuming 80 years life expectancy, all incurred at the year of death with years of life remaining discounted by 3% per year)	Years lost to disability plus years of life lost (one incurred in each year from year of death, assuming 80 years life expectancy)	Years lost to disability plus years of life lost discounted at 3%; disability weights used to calculate years lost to disability were obtained from the Global Burden of Disease Study	Years lost to disability plus years life of lost (life expectancy at death calculated from West Level 26 life table)
Average treatment and care unit costs per person on ART (US$)	$204	$191 (South Africa), $157 (Malawi), and $250 (Zimbabwe)	$140	$249	$197 (treatment only)

STI=sexually transmitted infections. ART=antiretroviral therapy. PSPO=parallel simultaneous perturbation optimisation. MSM=men who have sex with men. PWID=people who inject drugs. PrEP=pre-exposure prophylaxis. MTCT=mother-to-child HIV transmission. PEP=post-exposure prophylaxis. FSW=female sex workers. VMMC=voluntary medical male circumcision. DALY=disability adjusted life-year.

**Table 2:** Description of setting scenarios at start of 2021

		**South Africa**				**Malawi**		**Zimbabwe**		**Range of setting scenarios (HIV Synthesis, median [90% range])**
		Goals-ASM	Optima HIV, mean (90% range)	EMOD, mean (90% range)	Thembisa, mean (95% range)	Goals-ASM	Optima HIV, mean (90% range)	Goals-ASM	Optima HIV, mean (90% range)	
HIV prevalence*, %		19·5%	15·9% (12·3–19·7)	19·3% (18·3–20·4)	18·6% (18·2–19·0)	7·9%	9·8% (7·3–12·1)	11·3%	12·6% (10·9–14·1)	7·1% (2·0–20·0)
HIV incidence per 100 person-years*		0·73	0·48 (0·31–0·62)	0·93 (0·89–1·00)	0·57 (0·53–0·61)	0·19	0·31 (0·19–0·41)	0·25	0·25 (0·16–0·27)	0·36 (0·06–1·27)
Proportion of all HIV positive people with viral load <1000 copies per mL†‡, %		58·4%§	56·1% (55·2–58·0)	63·0% (62·8–63·3)	66·5% (65·6–67·9)	74·9%§	66·0% (65·4–73·0)	71·3%§	72·8% (72·4–80·0)	71·6% (45·2–84·3)
Proportion of all HIV positive people on ART†, %		73·9%	66·8% (59·9–71·1)	72·4% (72·2–72·7)	70·5% (69·5–71·5)	85·8%	81·9% (78·9–88·5)	86·0%	90·4% (85·1–97·6)	82·2% (62·1–92·7)
Proportion of men ever circumcised¶, %										
	15–49 years	67·7%	53·4% (52·8–53·9)	52·3% (52·1–52·6)	61·2%‖	33·7%	39·8% (39·1–40·6)	24·4%	40·6% (40·0–41·1)	67·2% (21·4–94·1)
	15–19 years	70·7%	80·6% (80·0–81·1)	42·0% (41·6–42·4)	64·6%‖	38·4%	38·4% (38·1–38·7)	43·7%	45·1% (44·9–45·2)	80·0% (18·8–95·8)
	20–24 years	80·2%	80·6% (80·0–81·1)	54·7% (54·3–55·2)	73·2%‖	35·8%	45·5% (45·1–45·8)	29·5%	50·3% (50·0–50·6)	79·7% (24·3–96·3)
	25–29 years	77·2%	40·5% (40·2–40·8)	58·3% (57·8–58·8)	67·2%‖	33·0%	49·2% (48·4–50·1)	18·2%	49·5% (48·8–50·2)	71·5% (23·6–95·3)
	30–34 years	68·6%	40·5% (40·2–40·8)	56·2% (55·8–56·6)	57·9%‖	31·1%	49·8% (48·6–51·1)	13·5%	49·5% (48·8–50·2)	63·9% (20·6–92·0)
	35–39 years	60·1%	40·5% (40·2–40·8)	53·4% (52·9–53·7)	54·3%‖	30·2%	33·0% (32·2–33·9)	12·3%	17·7% (17·3–18·1)	56·6% (19·2–93·5)
	40–44 years	54·9%	40·5% (40·2–40·8)	50·8% (50·4–51·3)	53·7%‖	29·7%	23·7% (23·2–24·3)	12·2%	17·7% (17·3–18·1)	53·7% (17·6–93·3)
	45–49 years	52·7%	40·5% (40·2–40·8)	50·2% (49·6–50·8)	54·3%‖	29·1%	19·0% (18·6–19·4)	12·5%	17·7% (17·3–18·1)	54·0% (17·5–93·5)

Data are mean or median (90% or 95% range). Expanded table with sex breakdown is shown in the appendix (pp 4–5). PHIA=Population-based HIV Impact Assessment. VMMC=voluntary medical male circumcision.

* Age 15–49 years.

† Age 15–64 years.

‡ Outputs shown might not reflect the latest PHIA results at time of print. However, any differences in viral suppression are unlikely to impact on conclusions (see appendix [p 8] for association between viral suppression and cost-effectiveness of VMMC).

§ Inputted into the model.

¶ Including non-medical and traditional circumcision, where this is full circumcision.

‖ Uncertainty in male circumcision parameters was not considered in Thembisa.

### Models

In the Optima HIV model (modelling South Africa, Malawi, and Zimbabwe), the most recent baseline rates of traditional male circumcision were informed by the Malawi Demographic and Health Survey (DHS) 2015–16, South African National HIV Prevalence, Incidence, Behaviour and Communication Survey (SABSSM V) 2017, and Zimbabwe Population-based HIV Impact Assessment (ZIMPHIA) 2015–16, adjusted to exclude reported VMMCs over preceding years, and assumed to remain constant in future years.^20^, ^21^, ^22^ The number of VMMCs performed annually was informed by programmatic reporting from each country. The overall proportion of annually circumcised men by 5-year age group was determined in the model through a combination of rates of traditional circumcision and the number of VMMCs, as well as population growth and ageing. The Optima HIV model version 2.10.1 is available online.

In EMOD (modelling South Africa), baseline rates of traditional male circumcision were obtained from national surveys.^23^, ^24^, ^25^ Traditional circumcision (adjusted for data being self-reported and considering some individuals might not be fully circumcised) is assumed to occur before sexual debut in 42% of men nationally. VMMCs, in contrast, can occur either before or after sexual debut according to age-stratified estimates provided by programmatic data from 2010 to 2018. For 2019–20, VMMC is distributed using the 2017–22 National Strategic Plan, which targets a cumulative 3 million VMMCs to be performed between 2017 and 2022. EMOD version 2.5 is available online. Detailed model description, parameter definitions, and usage instructions are available on the model's websites and elsewhere.^15^, ^26^, ^27^

In Thembisa (modelling South Africa), baseline rates of male circumcision (which include traditional circumcision) are specified by age; these are assumed to be constant over time and represent the fraction of men circumcised in the absence of campaigns promoting VMMC as an HIV prevention strategy. Age-specific rates of VMMC uptake as a result of campaigns are estimated from reported annual numbers of VMMC operations by age. A more detailed description of the model and the male circumcision assumptions is provided elsewhere (Thembisa version 4.3).

In Goals-ASM (modelling focused on Malawi, South Africa, and Zimbabwe, and also including Botswana, Eswatini, Lesotho, Mozambique, Namibia, Rwanda, Uganda, and Zambia), male circumcision is represented in terms of coverages by year for 5-year age groups in those aged 15 years to 49 years. Coverage trends pre-2020, including medical and traditional circumcisions, were estimated from DHS, AIDS Indicators Surveys (AIS), Population-based HIV Impact Assessment (PHIA), and other comparable survey data. For years 2021 and later, coverages were calculated under different assumptions for the two scenarios. Under the continuation of the VMMC scenario, circumcision coverage is increased from the baseline values in 2020 to 90% in 2025 for ages 15–29 years only (VMMC scale-up in these age groups). Starting in 2026 for ages 15–29 years, and in 2021 for ages 30–49 years, VMMC is discontinued, and coverages decline to their traditional levels (pre-VMMC coverage by age), accounting for ageing. Under the no further VMMC scenario, coverage immediately begins to decline in 2021 to baseline (pre-VMMC programme) levels for all age groups, again accounting for ageing. The number of VMMCs performed in each age group by year is calculated using a simple demographic model to produce the coverage trends in each scenario, accounting for ageing, death, and ongoing circumcisions at pre-2008 rates (which are assumed constant).

In HIV Synthesis, rather than calibrating the model to a specific setting or country, a range of setting scenarios (n=200) were generated. We aim to represent the diversity of the HIV epidemic and programme characteristics across and within countries in sub-Saharan Africa, including VMMC prevalence across the region. Age-specific probabilities for VMMC uptake are sampled to reflect differences in VMMC according to age and calendar year.

Models compared two policy options going forward: (1) continuation of VMMC in men aged 15 years and older as described here for each model for 5 years from July 1, 2021 (note for those younger than 15 years there is no further VMMC in Goals, Optima, HIV-Synthesis, and EMOD, while in Thembisa [modelling South Africa] this continues), and (2) no further VMMC in any age group after July 1, 2021. This is because WHO and PEPFAR recently published guidance to discontinue circumcision in those younger than 15 years for safety and ethical concerns. Although circumcisions continue for this group in some countries, most countries have adopted this policy.^1^, ^28^

### Analyses

Results are expressed as the mean difference between the two policies (VMMC continuation for 5 years *vs* no further VMMC) over three time horizons: 5 years, 20 years, and 50 years. 90% ranges are also calculated to convey uncertainty across runs. Outputs include HIV incidence, HIV prevalence, HIV mortality, the percentage of infections averted, and the number of VMMCs needed to prevent one HIV infection. Disability-adjusted life-years (DALYs) incurred, compared between policy options over 5 years, 20 years, and 50 years, were used as our main measure of effectiveness for our cost-effectiveness calculations. Although DALYs were similarly defined between models, specific calculations of DALYs varied slightly between models (table 1).

Both VMMC-specific costs and total HIV-care costs (including HIV testing, subsequent ART provision, and associated care) accrued over the time horizons were estimated. Inputs regarding the average cost of ART provision and associated care per diagnosed person on ART are shown in table 1. These costs could not be aligned between models as each model used different costing techniques which were integral to the structure of each model.

Cost-effectiveness analyses were done from a health-care provider perspective; costs and health outcomes were discounted to 2020 US$ values at 3% per annum. Results are presented as incremental cost per infection-averted and per DALY-averted.

There is variable evidence regarding the unit costs of VMMC.^29^ VMMC programmes were mainly established through donor-led initiatives and are still not well integrated into national-led (eg, Ministry of Health) programmes. Therefore, some personnel costs (including management and supervision) are likely to be higher than if VMMC was delivered within national programmes, and programme development and start-up costs are not generally captured. However, although the average service provision costs of VMMC can range from US$30 to US$200 (personal communication Heather Watts, PEPFAR), PEPFAR has capped expenditure at US$90 per circumcision in the budgets for the Country Operation Plans 2021 (PEPFAR 2021). The US$90 cap is aligned with the South African VMMC government fee of US$86·47. Hence, for these projections, we have assumed the fully loaded (total cost of delivering VMMC per person) average cost of VMMC to be US$90 in all settings. In addition, sensitivity analyses using average costs of US$60 and US$120 per VMMC were also performed.

Cost-effectiveness results are compared with a cost-effectiveness threshold representing the health which could be generated with other uses of the required resources. We use a cost-effectiveness threshold of US$500 per DALY. Country-specific thresholds are generally uncertain, and while higher thresholds have been recommended in, for example, South Africa,^30^, ^31^ US$500 per DALY averted is likely to be at the upper end, based on evidence concerning how budgets would otherwise be used in most lower-income settings in sub-Saharan Africa^32^ and, in Malawi and Zimbabwe in particular, reflects the extensive donor funding for HIV.

To determine the total health impact from VMMC, we also calculate net DALYs. This measure takes into account both the difference in actual DALYs averted and the difference in DALYs arising as a result of the difference in cost between the scenarios. This difference in cost is converted into a difference in DALYs through the use of the cost-effectiveness threshold. Thus, the difference in net DALYs between the two scenarios is given by the difference in DALYs plus the difference in costs divided by the cost-effectiveness threshold. Therefore, net DALYs is a measure of the health effects of an intervention encompassing the full implications of the intervention being delivered across the health-care system.

In our sensitivity analyses, we considered the possibility that ART coverage could be lower in 2041 than in 2021, based on concerns over risks of health-care delivery disruptions due to challenges such as heavily reduced global funding, conflict, natural disasters, pandemics (such as the ongoing COVID-19 pandemic), or shifts in health-care priorities. However, the degree to which ART coverage could decline is uncertain. We evaluated a scenario including a steady 20% decline in ART coverage by 2040 from 2021 levels solely to illustrate the effect of a potentially lower future treatment coverage.

### Role of the funding source

The funder of the study had no role in study design, data collection, data analysis, data interpretation, or writing of the report.

## Results

Characteristics of the modelled populations in 2021, stratified by country where applicable, are shown in table 2. Comparable PHIA data for each output are shown in the appendix (pp 1–3). Characteristics for the same countries generated by different models are broadly in line with each other, and, within the HIV Synthesis model 90% ranges across setting scenarios. The estimate with the most variability within any one setting was HIV incidence: in South Africa, this ranged from 0·48 per 100 person-years (Optima HIV) to 0·93 per 100 person-years (EMOD). Predicted characteristics of HIV epidemics in 2041 assuming continuation of VMMC for 5 years are shown in table 3. In particular, estimates for HIV prevalence and HIV incidence are similar across models modelling the same countries (South Africa incidence range of 0·19–0·57 per 100 person-years, Malawi incidence range of 0·09–0·16 per 100 person-years, and Zimbabwe incidence range of 0·11–0·15 per 100 person-years). However, there are some differences in the percentage of people living with HIV with undetectable viral load in 2041; in South Africa, this ranges from 55·5% to 72·8%, and in Malawi from 69·2% to 79·2%.

**Table 3:** Modelled projected outputs in 2041 assuming continuation of VMMC for 5 years from 2021 under current ART assumptions

		**South Africa**				**Malawi**		**Zimbabwe**		**Setting scenarios (median HIV Synthesis [90% range])**
		Goals-ASM	Optima HIV, mean (90% range)	EMOD, mean (90% range)	Thembisa, mean (95% range)	Goals-ASM	Optima HIV, mean (90% range)	Goals-ASM	Optima HIV, mean (90% range)	
HIV prevalence*, %		6·3%	9·3% (7·7–14·9)	10·5% (9·9–11·3)	7·6% (7·2–8·0)	2·5%	4·4% (4·1–4·8)	3·4%	6·8% (6·1–10·5)	3·3% (0·6–14·3)
HIV incidence per 100 person-years*		0·30	0·28 (0·17–0·36)	0·57 (0·52–0·61)	0·19 (0·17–0·22)	0·09	0·16 (0·10–0·20)	0·11	0·15 (0·08–0·15)	0·26 (0·03–1·23)
Proportion of all HIV positive people with viral load <1000 copies per mL†, %		65·4%	55·5% (55·0–56·5)	67·9% (67·8–68·7)	72·8% (72·4–73·2)	79·2% (67·6–83·6)	69·2% (66·4–71·0)	74·8% (65·3–78·4)	74·6% (73·2–75·1)	74·8% (53·4–86·0)
Proportion of all HIV positive people on ART†, %		74·9 %	66·3% (60·1–71·4)	80·1% (79·8–80·4)	76·8% (76·4–77·1)	87·4%	81·5% (78·7–88·3)	87·8%	90·3% (85·0–96·4)	93·1% (81·5–97·3)
Proportion of men ever circumcised‡, %										
	15–49 years	63·3%	50·6% (50·2–51·1)	61·4% (61·2–61·5)	68·4%§	48·0%	30·1% (29·9–30·4)	42·0%	32·6% (32·4–32·7)	46·0% (17·6–92·9)
	15–19 years	25·7%	39·7% (39·6–39·9)	8·0% (7·8–8·2)	20·2%§	16·4%	15·1% (15·1–15·2)	7·3%	11·6% (11·6–11·6)	10·0% (5·0–90·0)
	20–24 years	38·7%	39·7% (39·6–39·9)	23·0% (22·7–23·3)	31·4%§	18·8%	18·7% (18·6–18·8)	9·0%	17·5% (17·5–17·5)	10·2% (4·6–90·1)
	25–29 years	46·4%	53·9% (53·4–54·5)	40·7% (40·4–41·1)	80·8%§	20·3%	25·2% (25·0–25·3)	10·3%	38·2% (37·9–38·3)	15·4% (5·9–90·2)
	30–34 years	90·0%	53·9% (53·4–54·5)	95·1% (95·0–95·3)	96·0%§	90·0%	34·5% (34·2–34·9)	90·0%	38·2% (37·9–38·3)	74·4% (20·7–95·3)
	35–39 years	90·0%	53·9% (53·4–54·5)	98·4% (98·3–98·5)	94·8%§	90·0%	43·8% (43·3–44·3)	90·0%	47·6% (47·0–47·9)	83·6% (30·5–96·9)
	40–44 years	90·0%	53·9% (53·4–54·5)	97·2% (97·0–97·4)	89·5%§	90·0%	49·7% (49·0–50·5)	90·0%	47·6% (47·0–47·9)	82·1% (31·6–96·8)
	45–49 years	78·6%	53·9% (53·4–54·5)	76·5% (76·1–76·9)	78·0%§	51·1%	52·0% (51·1–53·1)	40·2%	47·6% (47·0–47·9)	77·3% (28·8–95·9)

Data are mean or median (90% or 95% range). ART=antiretroviral therapy. VMMC=voluntary medical male circumcision.

* Age 15–49 years.

† Age 15–64 years.

‡ Including non-medical and traditional circumcision, where this is full circumcision.

§ Uncertainty in male circumcision parameters was not considered in Thembisa.

Figure A and B show differences in the percentage of men ever circumcised and HIV incidence in men according to policy options and over the three time horizons. As expected (given the intervention is restricted to 5 years), the difference in the percentage of men ever circumcised is higher over shorter time horizons. HIV incidence in men is lower with the continuation of VMMC for 5 years in all settings over all time horizons. Modest differences in mortality rates are also seen on average over 20-year and 50-year time horizons with the continuation of VMMC for 5 years compared with no further VMMC (figure C). Figure D shows the percentage of infections averted in the entire population with the continuation of VMMC for 5 years versus discontinuation of VMMC. Over a 50-year time horizon, up to 12% of infections were predicted to be averted with the continuation of VMMC for 5 years in South Africa (4·3% with Optima HIV to 12·3% Thembisa), up to 15% in Malawi (7·0% with Optima HIV to 15·3% with Goals-ASM), and up to 14% in Zimbabwe (4·1% with Optima HIV to 13·9% Goals-ASM).

**Figure:**
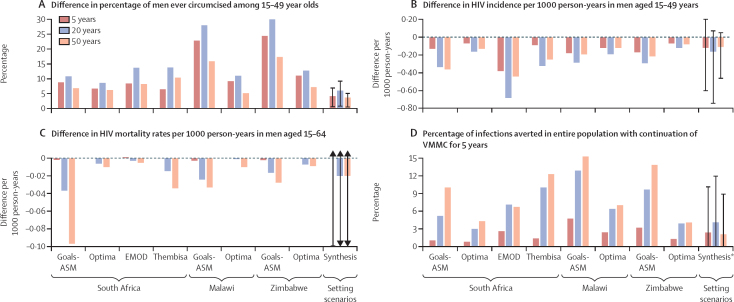
Absolute differences in mean percentage of men ever circumcised, HIV incidence, HIV mortality, and percentage of infections averted across time horizons comparing continuation of VMMC for 5 years with no further VMMC across time horizons VMMC=voluntary medical male circumcision. Error bars show 90% range. *Lower limit for 90% range for HIV Synthesis is “infections not averted”.

Although the number of VMMCs needed to avert one infection varied across models for the same setting, there was a clear and non-overlapping rank order across the three countries. The least number of VMMCs needed to prevent one undiscounted HIV infection over a 50-year time horizon was in South Africa (two according to Goals-ASM, six according to EMOD, and seven according to Thembisa and Optima HIV). In Malawi, this ranged from nine (Optima HIV) to ten (Goals-ASM), and in Zimbabwe, from ten (Goals-ASM) to 23 (Optima HIV). Over the range of setting scenarios considered by HIV Synthesis, the median number of VMMCs needed to prevent one HIV infection was nine across a 50-year time horizon.

Table 4 shows the cost-effectiveness results. The mean total annual HIV programme cost of continuation of VMMC for 5 years was lower than no further VMMC over the course of a 50-year time horizon according to all models in South Africa (four models) and Malawi (two models), according to one out of two models (Goals-ASM) in Zimbabwe and in 60% of setting scenarios according to the Synthesis model. In addition, the mean total ART cost was also lower in the continuation of VMMC for 5 years scenario according to all models in all settings over a 50-year time horizon (appendix pp 5–7).

**Table 4:** Costs in US$ in 2021 and DALYs stratified by model, setting, and time horizon

	**South Africa**				**Malawi**		**Zimbabwe**		**Setting scenarios (HIV Synthesis)**
	Goals-ASM	Optima HIV	EMOD	Thembisa	Goals-ASM	Optima HIV	Goals-ASM	Optima HIV	
Difference in total costs comparing continuation of VMMC for 5 years to no further VMMC (US$ millions per year)*									
5 years	+9·0	+7·7	+10·0	+5·6	+32·9	+11·7	+32·3	+12·7	+6·1 (+0·7 to +9·9)
20 years	+1·3	+1·6	+1·0	+0·1	+6·4	+2·5	+6·4	+3·5	+1·0 (−0·2 to +2·2)
50 years	−2·2	−0·8	−2·0	−2·3	−0·7	−0·2	−0·5	+0·9	−0·2 (−2·1 to +0·8)
**Cost in US$ per infection averted (discounted difference in costs or discounted infections averted)**									
5 years	15 627	27 166	7751	14 028	33 030	20 247	40 089	51 794	9132 (976, infections not averted)
20 years	729	1836	202	52	2917	1990	3416	5793	1218 (infections averted and costs saved [VMMC dominates†], infections not averted)
50 years	Infections averted and costs saved (VMMC dominates†)	Infections averted and costs saved (VMMC dominates)	Infections averted and costs saved (VMMC dominates)	Infections averted and costs saved (VMMC dominates)	Infections averted and costs saved (VMMC dominates)	Infections averted and costs saved (VMMC dominates)	Infections averted and costs saved (VMMC dominates)	1903	Infections averted and costs saved (VMMC dominates [infections averted and costs saved, infections not averted])
**Difference in DALYs comparing no VMMC with continued VMMC (DALYs for no VMMC–DALYS for continued VMMC; mean per year over time horizon, 3% discount rate)**									
5 years	2219	277	535	125	1009	130	534	45	−66‡ (−3155 to 3753)
20 years	27 516	3898	13 555	9133	7811	571	3884	662	794 (−2702 to 4946)
50 years	47 104	6796	42 858	16 420	9562	2075	5244	910	1041 (−954 to 4491)
**Cost-per-DALY averted for continued VMMC relative to no further VMMC**									
5 years	15 612	114 719	10 657	202 375	33 734	98 337	48 829	220 319	DALYs not averted (1034, DALYs not averted)
20 years	180	1522	265	47	845	4480	1339	3585	1245 (cost saving [$0·2 per year], DALYs not averted)
50 years	VMMC dominates†	VMMC dominates	VMMC dominates	VMMC dominates	VMMC dominates	VMMC dominates	VMMC dominates	616	60 (VMMC dominates, DALYs not averted)§
**Difference in net DALYs comparing continued VMMC *vs* no VMMC assuming CET of US$500 per DALY averted (mean per year over time horizon)**									
5 years	67 056 additional	63 186 additional	114 075 additional	50 600 additional	67 039 additional	25 506 additional	51 615 additional	19 801 additional	12 096 additional (20 670 additional, 1443 additional)
20 years	17 160 additional	7971additional	6370averted	8283averted	5394 additional	4548 additional	6517 additional	4083 additional	1285 additional (−5039 additional, 4184 averted)
50 years	64 020 averted	11 552 averted	72 379 averted	42 956 averted	11 045 averted	2259 averted	6084 averted	210 additional	1267 averted (1822 additional, 7792 averted)

Assumed unit cost of VMMC is US$90. Costs and DALYs are per 10 million adults aged 15–64 years. Costs and DALYs discounted at 3% per year. Expanded table including total programme costs and total ART costs is shown in the appendix (pp 5–7). DALYs=disability-adjusted life-years. VMMC=voluntary medical male circumcision. CET=cost-effectiveness threshold. ART=antiretroviral therapy.

* Optima HIV costs include only VMMC and ART costs.

† Continuation of VMMC dominates no VMMC, offering both health benefits (infections and DALYs averted) and reduced costs.

‡ Negative DALYs are a result of stochastic variation; no negative effect of VMMC is assumed.

§ Assuming maximum difference in costs and one infection averted for setting scenarios in which infections are not averted.

Lower programme and ART costs led to the continuation of VMMC being cost-saving as well as averting infections for all models in all countries, except for Optima HIV in Zimbabwe where the cost-per-infection averted was estimated to be US$1903 over a 50-year time horizon. In addition, DALYs were averted with the continuation of VMMC in all models (for HIV Synthesis, the median across runs was used for this calculation), with health benefits accumulating substantially over 50 years.

The continuation of VMMC resulted in cost savings and averted DALYs compared with no further VMMC for all models in South Africa and Malawi by 50 years. And the continuation of VMMC was cost-effective in 68% of setting scenarios modelled by HIV Synthesis (median cost per DALY averted of US$60—ie, lower than the cost-effectiveness threshold of US$500). In Zimbabwe, while Goals-ASM found continuation of VMMC to be cost-saving and averting DALYs, Optima HIV estimates for the cost per DALY averted was US$616, which was slightly higher than the adopted cost-effectiveness threshold of $500, indicating continuation of VMMC would not be cost-effective in this context. This difference primarily relates to Optima HIV projecting a more rapid decline in future HIV incidence between 2020 and 2030 than Goals (appendix p 20), allowing less scope for VMMC to provide additional benefit. Other factors such as differences in demographic projections, costs of clinical care, and the cumulative effect over time of small differences between models—ie, diagnosis, treatment initiation, and viral suppression rates—could also have impacted these outputs.

Continuation of VMMC for 5 years was cost-effective and cost-saving according to Goals-ASM in most other African countries modelled: Eswatini, Lesotho (cost-saving), Mozambique (cost-saving), Namibia (cost-saving), Uganda (cost-saving), and Zambia. In Botswana, despite similar HIV incidence to other settings modelled, such as Uganda (0·15 per 100 person-years, where VMMC was found to provide cost savings), the continuation of VMMC was not shown to be cost-effective. Although higher VMMC coverage could explain this difference to an extent (50% in Uganda *vs* 43% in Botswana), population growth estimates differ considerably between these two settings; population size, and hence new HIV infections, is predicted to increase considerably in Uganda, whereas it is expected to be relatively stable in Botswana, which is likely to be the key driver of differences in cost-effectiveness. The continuation of VMMC was also not cost-effective in Rwanda. HIV incidence in Rwanda was estimated to be the lowest amongst all countries considered at 0·01 per 100 person-years in 2021 and was not predicted to change considerably by 2041.

Table 5 shows the percentage of scenarios in which VMMC was cost-effective according to 2021 HIV incidence rates as estimated by the Synthesis model. Continuation of VMMC was cost-effective in 62% of scenarios in which incidence was less than 0·1 per 100 person-years. This value increased to 95% for scenarios in which incidence was greater than 1·0 per 100 person years.

**Table 5:** HIV Synthesis—relationship between incidence and cost-effectiveness in 200 scenarios in 2021 per 100 person-years

	**Number of setting scenarios**	**Number of VMMCs needed to avert one HIV infection (median [90% range])**	**Percent of scenarios in which VMMC is cost-effective**
0·00–0·10	21	20 (2, infections not averted)	62%
0·11–0·20	35	16 (4, infections not averted)	57%
0·21–0·30	30	10 (3, infections not averted)	73%
0·31–0·50	49	8 (3, infections not averted)	88%
0·51–1·00	46	6 (1, infections not averted)	85%
>1·00	19	6 (1, infections not averted)	95%

VMMC=voluntary medical male circumcision.

In the sensitivity analyses, where the unit cost of VMMC was assumed to be US$60 rather than US$90, other than in Rwanda, the continuation of VMMC for 5 years was shown to be cost-effective (with larger cost savings than the primary analysis) in all settings by all models, including in Zimbabwe by Optima HIV (appendix pp 9–10) and in Botswana by Goals-ASM. Even when assuming a higher unit cost of VMMC of US$120, continuation of VMMC for 5 years was cost-saving in South Africa (according to all models), cost-effective in Malawi (two models, cost-saving according to Goals-ASM), and on average across a range of setting scenarios. In Zimbabwe, continuation of VMMC at a unit cost of $120 was not cost-effective according to Optima HIV but was still cost-effective according to Goals-ASM (appendix pp 11–12).

Assuming 20% lower ART coverage in 2041 (outputs for 2041 are shown in the appendix [p 13]), VMMC was cost-effective over a 50-year time horizon in all settings using all models, with increased cost savings compared with the primary analysis in all settings (appendix pp 14–15). Finally, when DALYs were discounted by 5% rather than 3%, continuation of VMMC for 5 years remained cost-effective on average across a range of setting scenarios according to HIV Synthesis, in South Africa and Malawi for all models, and Zimbabwe according to Goals-ASM but not according to Optima HIV (appendix pp 16–17).

## Discussion

Despite differences in projected HIV incidence and other differences in the structure, assumptions, and predicted epidemics across models, there was agreement across models that the continuation of VMMC for 5 years in adults aged over 15 years was cost-effective in almost all settings and cost-saving over sufficiently long time horizons. VMMC would provide cost-saving over 50 years according to all models in South Africa and Malawi and according to Goals-ASM in Zimbabwe. Over a range of setting scenarios, the continuation of VMMC for 5 years was cost-effective when analysed over 50 years in three-quarters of setting scenarios in which incidence was between 0·2 and 0·3 per 100 person-years in those aged 15–49 years in 2021. Continuation of VMMC remained cost-effective in 62% of setting scenarios with very low HIV incidence (<0·1 per 100 person-years). Continuation of VMMC for 5 years was also predicted to lower HIV incidence over all time horizons and have a modest impact on male HIV mortality rates over 20-year and 50-year time horizons.

We chose to compare the discontinuation of VMMC with the continuation of VMMC for 5 years in men aged 15 years or older given recommendations to stop VMMC in boys aged 10–14 years due to safety concerns.^1^, ^28^ 5 years was chosen as the intervention period and 50 years as the analysis period so the full effects of VMMC could be captured; if we were to look at the continuation of VMMC across a 50-year time horizon, the benefits of VMMCs (eg, impact on HIV incidence) performed later in the 50 years would not be captured in the analyses, whereas shorter analysis horizons would not capture the full benefit of VMMCs on life expectancy in settings with high levels of ART coverage and viral suppression. Absolute annual reductions in HIV incidence with the continuation of VMMC were similar across settings and more pronounced when considering a 20-year time horizon than a longer time horizon of 50 years. This effect is expected given that the 15–20-year period after VMMC is when we would expect to see the most benefit of VMMCs in terms of HIV incidence, as HIV incidence is expected to decline over time. Other modelling studies, while focusing on age targeting, have also predicted the potential benefit of VMMC over future time horizons.

Stegman and colleagues showed that the greatest impact on HIV incidence and the most cost-effective strategy in Namibia over the next 15 years would be to circumcise men aged 15–24 years, although they noted over longer time horizons, reductions in HIV incidence would be considerable if younger men (aged 10–14 years) were circumcised.^33^ Using the same model, Kripke and colleagues, focusing on age targeting of VMMC, showed that the largest reduction in HIV incidence over 15 years was predicted in South Africa and Malawi if VMMC was targeted to men aged 15–24 years.^6^, ^34^

Given that we have only considered a 5-year intervention and numbers of VMMCs performed in each country were not standardised across models for the same country, our impact results are conservative. We expect larger differences in HIV incidence and mortality rates if the intervention were continued for more than 5 years. However, based on these analyses, we cannot say if VMMC will continue to be cost-effective after 5 years; the future cost associated with maintaining high VMMC coverage in a growing population needs to be considered and weighed against other potentially cheaper options, such as routine neonatal circumcision, which to date have not been the focus of prevention programmes. It can be argued that not investing in neonatal circumcisions presents a missed opportunity and this needs to be given further consideration.^35^ Therefore, it is likely to be important for us to update this analysis in 5 years to see if VMMC is predicted to remain cost-effective.

When compared with no further VMMC, we found up to 15% of HIV infections were predicted to be averted over the course of 50 years with the continuation of VMMC for 5 years. Kripke and colleagues showed HIV infections were averted in a range of VMMC programme scenarios in Malawi over a considerably shorter time horizon (15 years), with the scale-up of VMMC to 80% in those aged 15–49 years being associated with the highest number of infections averted.^7^ Other modelling studies have also shown similar results. For example, McGillen and colleagues used results from three models (including Goals and EMOD) calibrated to VMMC coverage in Zimbabwe to predict the number of HIV infections averted. It was estimated that up to 12 000 infections were averted in 2016 with the VMMC programme that was in place since 2009 in Zimbabwe, rising to 170 000 in 2030 if VMMC targets are maintained.^36^ WHO reported an estimated 340 000 infections averted between 2008 and 2019 in the 15 WHO VMMC priority countries of east and southern Africa based on modelling methods used by McGillen and colleagues.^4^ This included 260 000 infections in men and 75 000 in women (due to reduced secondary transmission from men). A modelling study that included results from Goals-ASM, Thembisa, and EMOD estimated that VMMC had averted up to 83 000 HIV infections from 2010 to 2017 and had already had a modest impact on HIV incidence in South Africa.^12^

We have shown that VMMC is more likely to be cost-effective in countries and settings with high HIV incidence, although even in settings with very low HIV incidence, VMMC was shown to be cost-effective in most settings using HIV Synthesis. Goals-ASM showed similar findings across the range of countries they modelled. This is in line with the magnitude of cost-saving being higher in South Africa with the continuation of VMMC for 5 years than in Malawi and Zimbabwe, where HIV incidence is lower. The cost-effectiveness of VMMC is also likely to depend on trajectories of future HIV incidence. Similar to baseline incidence, settings with high HIV incidence in 2041 were more likely to be associated with cost savings from the continued VMMC scenario than settings with lower HIV incidence in 2041. Another key factor in determining whether an intervention such as VMMC is cost-effective is future population growth. A considerable increase in population is likely to result in new HIV infections also increasing. As shown by Goals-ASM, despite similar baseline incidence in Botswana and Uganda, the continuation of VMMC was only cost-effective in Uganda. Uganda's population is expected to increase considerably over the time horizon analysed (3·3% annual growth rate, to 89 million in 2050 according to World Population Review). Conversely, the population in Botswana is predicted to grow at a slower rate (1·8%, to 3·5 million in 2050).

Our sensitivity analyses revealed that VMMC was more cost-effective in lower ART coverage scenarios. Hence, it can be seen as an insurance policy: in the event that ART coverage were to decline or HIV incidence were to increase for reasons such as population growth, there would be prevention measures in place to limit the extent of a resurgence in incidence. The negative consequences of lower ART coverage would likely outweigh almost all other disruptions to HIV services. As has been done throughout the COVID-19 pandemic, countries should continue prioritising keeping people on ART in the event of further disruptions to HIV services.^37^

Since these model results were produced, emerging data from Malawi suggest that higher levels of viral suppression have been achieved than those we have shown in our baseline table.^35^ However, we do not expect recalibrations to change our conclusions, particularly since we did not find viral suppression rates to be a predictor of scenarios being cost-effective (appendix p 8). Although we do attempt to convey uncertainty, our modelled predictions are reliant on the data available to inform the models. In these specific analyses, the unit cost of VMMC and HIV incidence (both baseline and future trajectories) directly affect whether the continuation of VMMC is cost-effective. Unit costs of VMMC are dependent on whether programmes are donor-led, on demand for VMMC, the required costs of any demand generation, and uptake of VMMC. If costs are largely fixed, then a lower uptake will result in a higher unit cost, likely leading to a less cost-effective programme. This can also have implications for future projections; costs could be incurred, but benefits might not be realised if VMMC uptake is low. We have shown in settings with high HIV incidence that VMMC can be cost-effective even when unit costs are as high as US$120 (costs might be higher, for example, when targeting middle-aged men, which requires more investment in mobilisation or incentives). Conversely, if unit costs were lower at US$60, VMMC is likely to be cost-effective in settings where incidence is as low as 0·19 per 100 person-years, such as Malawi (as modelled by Goals-ASM). There is also uncertainty around an appropriate threshold for cost-effectiveness. The US$500 per DALY averted might be reasonable if programmes are donor-funded. However, a lower value is likely to be more appropriate if programmes are domestically funded by ministries of health.

VMMC has benefits other than the direct benefits explored in these analyses. It is a safe procedure with few adverse events.^38^ Also, it decreases the risk of acquiring and transmitting many other sexually transmitted infections such as herpes simplex virus and human papillomavirus.^39^, ^40^ VMMC also indirectly benefits women; secondary HIV transmission to women from men is reduced;^4^ VMMC has also been shown to reduce the incidence of cervical cancer.^41^, ^42^ It is a low-cost intervention, and this is the key reason VMMC is cost-effective in most settings. It is important to recognise that national HIV incidence or VMMC estimates might not reflect regional estimates. Although subnational targeting in regions with high HIV incidence and low circumcision coverage is likely to result in VMMC being more cost-effective, we have shown here that even in regions with low incidence, VMMC remains a cost-effective HIV prevention method.

VMMC programmes have a beneficial population health impact. We have shown here that the continuation of VMMC programmes for 5 years, in men older than 15 years, has a net health benefit, is cost-effective, and in fact cost-saving in almost all HIV incidence settings considered in this study over the course of 50 years. Our results support the continuation of VMMC for at least the next 5 years.

**This online publication has been corrected. The corrected version first appeared at thelancet.com/lancetgh on February 24, 2023**

## Data sharing

This is a modelling study and hence no new primary data were collected for this study. Source code for the models can be found on the individual model websites.

## Declaration of interests

LB-M has received support for the present manuscript paid to her institution from the Bill & Melinda Gates Foundation (BMGF). AB has received grants or contracts from the US National Institutes of Health and Foundation for Innovative New Diagnostics (FIND), and consulting fees from Gates Ventures. FMC has received grants or contracts paid to her institution from Wellcome Trust, BMGF, Medical Research Council (MRC), UNICEF, and UNITAID. GM-R has received support for the present manuscript paid to her institution from BMGF, and grants or contracts paid to her institution from National Institutes of Health, FIND, BMGF, and United States Agency for International Development (USAID). MH has received support for the present manuscript paid to his institution from BMGF. KK has received support for the present manuscript paid to her institution from BMGF and a grant from UNAIDS to support modelling activities not directly related to this manuscript. VC has received support for the present manuscript paid to her institution from BMGF and grants paid to her institution from the UK Research and Innovation, UNITAID, National Institute for Health Research, USAID, MRC, and BMGF. VC has received consulting fees from WHO. AP has received support for the present manuscript paid to his institution from BMGF. All other authors declare no competing interests.
