# Supplementary material for: Cost-effectiveness of voluntary medical male circumcision for HIV prevention across sub-Saharan Africa: results from five independent models
Source: Lancet Glob Health. 2022 Dec 20;11(2):e244–55. doi: 10.1016/S2214-109X(22)00515-0 (PMC10005968; doi:10.1016/S2214-109X(22)00515-0)

THE LANCET
Global Health

Supplementary appendix

This appendix formed part of the original submission and has been peer reviewed. We post it as supplied by the authors.

Supplement to: Bansi-Matharu L, Mudimu E, Martin-Hughes R, et al. Cost-effectiveness of voluntary medical male circumcision for HIV prevention across sub-Saharan Africa: results from five independent models. *Lancet Glob Health* 2022; published online Dec 20. https://doi.org/10.1016/S2214-109X(22)00515-0.

Table A0: Observed data

Characteristic	observed data (from PHIAs unless otherwise stated)					
HIV prevalence All/male/female (age 15–49)	Cameroon	2017	3%/2%/5%	Namibia	2016	12%/8%/15%
	Cote d'Ivoire	2017	3%/1%/4%	South Africa		-
	Eswatini	2017	27%/19%/34%	Tanzania	2017	5%/3%/6%
	Ethiopia	2018	3%/2%/4%	Uganda	2016	6%/4%/8%
	Lesotho	2017	24%/19%/30%	Zambia	2016	11%/8%/14%
	Malawi	2016	10%/8%/12%	Zimbabwe	2016	13%/11%/16%
	Mozambique		11%			
HIV incidence<br><br>All/male/female age 15–49 (/100 person years)	Cameroon	2017	0·24/0·08/0·40	Namibia	2016	0·40/0·15/0·66
	Cote d'Ivoire	2017	-	South Africa		
	Eswatini	2017	1·28/0·85/1·73	Tanzania	2017	0·24/0·14/0·34
	Ethiopia	2018	-	Uganda	2016	0·39/0·31/0·47
	Lesotho	2017	1·19/1·05/1·31	Zambia	2016	0·64/0·28/1·00
	Malawi	2016	0·33/0·22/0·44	Zimbabwe	2016	0·44/0·30/0·57
Proportion of HIV positive people diagnosed<br><br>All/male/female (age 15–64)	Cameroon	2017	56%/51%/58%	Namibia	2016	79%/71%/83%
	Cote d'Ivoire	2017	37%/24%/43%	South Africa		
	Eswatini	2017	85%/78%/89%	Tanzania	2017	52%/45%/55%
	Ethiopia	2018	79%/70%/83%	Uganda	2016	66%/62%/69%
	Lesotho	2017	77%/71%/82%	Zambia	2016	66%/62%/68%
	Malawi	2016	63%/68%/76%	Zimbabwe	2016	73%/68%/76%

Proportion of diagnosed HIV+ people on ART<br><br>All/male/female<br><br>(age 15–64)	Cameroon	2017	93%/94%/93%	Namibia	2016	96%/95%/97%
	Cote d'Ivoire	2017	88%/71%/93%	South Africa		
	Eswatini	2017	89%/90%/88%	Tanzania	2017	94%/90%/95%
	Ethiopia	2018	97%/99%/96%	Uganda	2016	90%/87%/92%
	Lesotho	2017	92%/92%/92%	Zambia	2016	87%/88%/87%
	Malawi	2016	91%/89%/93%	Zimbabwe	2016	88%/88%/89%
Proportion of all HIV positive people with VL < 1000 copies/mL<br><br>All/male/female<br><br>(age 15–64)	Cameroon	2017	45%/43%/46%	Namibia	2016	77%/70%/82%
	Cote d'Ivoire	2017	40%/28%/46%	South Africa		
	Eswatini	2017	73%/68%/76%	Tanzania	2017	52%/42%/57%
	Ethiopia	2018	70%/67%/72%	Uganda	2016	60%/54%/63%
	Lesotho	2017	68%/63%/71%	Zambia	2016	59%/57%/60%
	Malawi	2016	68%/61%/73%	Zimbabwe	2016	60%/54%/64%
Of people on ART, proportion with VL < 1000<br><br>All/male/female<br><br>(age 15–64)	Cameroon	2017	80%/81%/80%	Namibia	2016	91%/90%/92%
	Cote d'Ivoire	2017	76%/65%/78%	South Africa		
	Eswatini	2017	92%/91%/92%	Tanzania	2017	87%/83%/89%
	Ethiopia	2018	88%/91%/86%	Uganda	2016	84%/82%/85%
	Lesotho	2017	88%/88%/88%	Zambia	2016	89%/88%/90%
	Malawi	2016	91%/90%/92%	Zimbabwe	2016	85%/83%/87%
% males ever circumcised						

15+/ 15–19/ 20–24/ 25–29/30–34/ 35–39/ 40–44/ 45–49<br><br>(SA 15+/15–24/ 25–34/35–44/45–54)	Eswatini	2017	**27·6**/ 38·3/ 31·6/ 29·0/ 25·8/ 27·9/ 23·0/ 18·4			
	Lesotho	2017	**67·6**/ 63·8/ 72·6/ 71·5/ 70·7/ 65·6/ 67·7/ 62·6			
	Malawi	2016	**25·3**/ 26·7/ 28·5/ 23·2/ 23·5/ 26·2/ 22·6/ 23·9			
	Namibia	2016	**38·5**/ 39·3/ 41·0/ 39·7/ 38·4/ 39·4/ 37·9/ 36·5			
	South Africa	2017	**61·6**/ 70·2/ 62·8/ 62·6/ 55·2			
	Tanzania	2017	**77·6**/ 77·2/ 82·1/ 81·5/ 78·7/ 76·9/ 76·1/ 77·0			
	Uganda	2016	**42·2**/ 43·7/ 49·6/ 44·6/ 44·2/ 37·5/ 35·8/ 34·4			
	Zambia	2016	**27·1**/ 30·7/ 33·1/ 30·3/ 23·4/ 22·6/ 21·0/ 21·3			
	Zimbabwe	2016	**14·1**/ 23·6/ 17·6/ 11·8/ 7·8/ 8·1/ 9·2/ 10·9			
Latest total number of VMMC according to UNAIDS 2019	Cameroon	2017	-	Namibia	2017	34942
	Cote d'Ivoire	2017	-	Rwanda	2014	327904
	Eswatini	2016	14316	South Africa	2017	572442
	Ethiopia	2016	23009	Tanzania	2017	885599
	Kenya	2014	286889	Uganda	2016	619082
	Lesotho	2014	26448	Zambia	2016	482183
	Malawi	2016	199399	Zimbabwe	2016	326012
	Mozambique	2015	311891			

Table A1: Description of setting scenarios at start of 2021

Setting	South Africa				Malawi		Zimbabwe		Range of setting scenarios
**Model**	**Goals-ASM**	**Optima HIV Mean (90% range)**	**EMOD Mean (90% range)**	**Thembisa**	**Goals-ASM**	**Optima HIV Mean (90% range)**	**Goals-ASM**	**Optima HIV Mean (90% range)**	**HIV-Synthesis Median (90%range)**
HIV prevalence (%)<br>All/male/female<br>(age 15–49)	19·5 /<br>12·3 /<br>26·6	15·9 (12·3, 19·7) /<br>11·4 (8·8, 14·2) /<br>20·5 (15·9, 25·2)	19·3 (18·3, 20·4) /<br>13·4 (12·8, 14·1) /<br>25·2 (23·8, 26·5)	18·6 /<br>12·2 /<br>25·0	7·9 /<br>5·5 /<br>10·2	9·8 (7·3, 12·1)/<br>7·8 (5·8, 9·6) /<br>11·8 (8·8, 14·5)	11·3 /<br>7·7 /<br>14·6	12·6 (10·9, 14·1) /<br>10·0 (8·6, 11·2) /<br>14·9 (12·9, 16·5)	7·1 (2·0, 20·0) /<br>5·3 (1·5, 14·8) /<br>8·7 (2·4, 23·6)
HIV incidence/100 person years<br>All/male/female<br>(age 15–49)	0·73 /<br>0·41 /<br>1·12	0·48 (0·31, 0·62) /<br>0·32 (0·21, 0·41) /<br>0·65 (0·42, 0·86)	0·93 (0·89, 1·00) /<br>0·66 (0·62, 0·72) /<br>1·24 (1·15, 1·34)	0·57 /<br>0·36 /<br>0·81	0·19 /<br>0·11 /<br>0·26	0·31 (0·19, 0·41) /<br>0·24 (0·14, 0·32) /<br>0·38 (0·23, 0·51)	0·25 /<br>0·16 /<br>0·34	0·25 (0·16, 0·27) /<br>0·17 (0·11, 0·18) /<br>0·32 (0·21, 0·34)	0·36 (0·06, 1·27) /<br>0·29 (0·05, 1·12) /<br>0·40 (0·07, 1·51)
% of all HIV positive people with VL < 1000 copies/mL<br>All/male/female<br>(age 15–64)	58·4[1] /<br>49·2 /<br>63·1	56·1 (55·2, 58·0) /<br>55·7 (55·1, 58·0 /<br>56·3 (55·2, 58·0)	63·0 (62·8, 63·3) /<br>58·5 (58·0, 58·8) /<br>65·5 (65·2, 65·8)	66·5 /<br>61·6 /<br>69·0	74·9[1] /<br>64·7 /<br>81·0	66·0 (65·4, 73·0) /<br>66·2 (64·8, 73·0) /<br>65·9 (65·2, 73·0)	71·3[1] /<br>63·4 /<br>75·8	72·8 (72·4, 80·0) /<br>72·0 (71·0, 80·0) /<br>73·3 (72·6, 80·0)	71·6 (45·2, 84·2) /<br>63·8 (34·4, 78·6) /<br>77·1 (51·9, 87·3)
% of all HIV positive people on ART<br>(age 15–64)	73·9 /<br>67·4 /<br>77·3	66·8 (59·9, 71·1)	72·4 (72·2, 72·7)<br>70·7 (70·3, 71·1)<br>73·4 (73·1, 73·7)	70·5 /<br>65·5 /<br>73·0	85·8 /<br>78·3 /<br>90·4	81·9 (78·9, 88·5)	86·0 /<br>81·8 /<br>88·5	90·4 (85·1, 97·6)	82·2 (62·1, 92·7)<br>76·2 (51·4, 89·8)<br>86·3 (68·4, 94·3)
% males ever circumcised[2]									
**15–49**	**67·7**	**53·4**	**52·3 (52·1, 52·6)/**	**61·2**	**33·7**	**39·8**	**24·4**	**40·6**	**67·2 (21·4, 94·1)**
15–19	70·7	80·6	42·0 (41·6, 42·4)/	64·6	38·4	38·4	43·7	45·1	80·0 (18·8, 95·8)
20–24	80·2	80·6	54·7(54·3, 55·2)/	73·2	35·8	45·5	29·5	50·3	79·7 (24·3, 96·3)
25–29	77·2	40·5	58·3 (57·8, 58·8)/	67·2	33·0	49·2	18·2	49·5	71·5 (23·6, 95·3)
30–34	68·6	40·5	56·2 (55·8, 56·6)/	57·9	31·1	49·8	13·5	49·5	63·9 (20·6, 92·0)
35–39	60·1	40·5	53·4 (52·9, 53·7)/	54·3	30·2	33·0	12·3	17·7	56·6 (19·2, 93·5)

Setting	South Africa				Malawi		Zimbabwe		Range of setting scenarios
Model	Goals-ASM	Optima HIV Mean (90% range)	EMOD Mean (90% range)	Thembisa	Goals-ASM	Optima HIV Mean (90% range)	Goals-ASM	Optima HIV Mean (90% range)	HIV-Synthesis Median (90%range)
40–49<br>45–49	54·9<br>52·7	40·5<br>40·5	50·8 (50·4, 51·3)/<br>50·2 (49·6, 50·8)	53·7<br>54·3	29·7<br>29·1	23·7<br>19·0	12·2<br>12·5	17·7<br>17·7	53·7 (17·6, 93·3)<br>54·0 (17·5, 93·5)

Table A2: Costs (US$) and Disability Adjusted Life Years (DALYs) stratified by model, setting and time horizon (costs and DALYs discounted at 3% per year). Assuming VMMC cost = $90

Setting	South Africa				Malawi		Zimbabwe		Setting scenarios
Model	Goals-ASM	Optima HIV	EMOD	Thembisa	Goals-ASM	Optima HIV	Goals-ASM	Optima HIV	HIV-Synthesis
Mean annual total programme costs (not just VMMC) per 10 million[1] adults aged 15–64 over time horizon (US$, millions per year) (No further VMMC/ continuation of VMMC for 5 years)[1]									
5 years	398/407	268/275	286/296	346/ 352	199/232	140/151	255/287	250/262	142 (50, 326) / 149 (54, 332)
20 years	254/255	230/232	249/250	265/ 266	159/165	116/119	193/200	221/225	113 (38, 272) / 114 (38, 274)
50 years	154/152	176/175	165/163	150/148	113/113	85/85	125/124	180/181	80 (24, 205) / 80 (25, 205)
Difference in total costs per 10 million adults aged 15–64 comparing continuation of VMMC for 5 years to no further VMMC (USD millions per year)									
5 years	+9	+7·7	+10·0	+5·6	+32·9	+11·7	+32·3	+12·7	+6·1 (+0·7, +9·9)
20 years	1·3	+1·6	+1·0	+0·1	+6·4	+2·5	+6·4	+3·5	+1·0 (-0·2, +2·2)
50 years	-2·2	-0·8	-2·0	-2·3	-0·7	-0·2	-0·5	+0·9	-0·2 (-2·1, +0·8)

Setting	South Africa				Malawi		Zimbabwe		Setting scenarios
**Model**	**Goals-ASM**	**Optima HIV**	**EMOD**	**Thembisa**	**Goals-ASM**	**Optima HIV**	**Goals-ASM**	**Optima HIV**	**HIV-Synthesis**
Total ART costs per 10 million adults aged 15–64: all treatment and care costs for people with HIV (including any explicit costing of CD4/VL testing etc.) and clinical care of people with AIDS conditions, but not the cost of HIV testing									
5 years	375/375	267/267	222/222	334/334	161/161	139/139	233/233	249/249	117 (35, 304) / 117 (35, 304)
20 years	237/236	230/229	186/183	256/255	127/126	116/115	175/174	221/221	91 (26, 251) / 91 (26, 251)
50 years	143/140	176/174	118/114	144/141	86/82	85/83	111/107	180/179	60 (15, 188) / 59 (15, 187)
Difference in total ART costs per 10 million adults aged 15–64 comparing continuation of VMMC to no further VMMC (USD millions per year)									
5 years	-0·1	-0·0	0·0	0·0	-0·1	-0·0	-0·1	-0·0	+0·1 (-0·3, +0·6)
20 years	-0·9	-0·7	-2·4	-1·2	-1·9	-0·8	-1·7	-0·4	-0·4 (-2·1, +0·3)
50 years	-3·1	-1·8	-3·9	-2·8	-4	-1·7	-3·8	-1·0	-0·8 (-2·8, +0·2)
Cost per infection averted (discounted difference in costs/(discounted infections averted)									
5 years	15,627	27,166	7,751	14,028	33,030	20,247	40,089	51,794	9132 (976, Infections not averted)
20 years	729	1,836	202	52	2,917	1,990	3,416	5,793	1218 (VMMC dominates, Infections not averted)
50 years	VMMC dominates	VMMC dominates	VMMC dominates	VMMC dominates	VMMC dominates	VMMC dominates	VMMC dominates	1,903	VMMC dominates (VMMC dominates, Infections not averted)
Difference in DALYs comparing no VMMC with continued VMMC (DALYs for no VMMC – DALYS for continued VMMC; mean per year over time horizon, 3% discount rate)									
5 years	2,219	277	535	125	1,009	130	534	45	-66 (-3155, 3753)

Setting	South Africa				Malawi		Zimbabwe		Setting scenarios
Model	Goals-ASM	Optima HIV	EMOD	Thembisa	Goals-ASM	Optima HIV	Goals-ASM	Optima HIV	HIV-Synthesis
20 years	27,516	3,898	13,555	9,133	7,811	571	3,884	662	794 (-2702, 4946)
50 years	47,104	6,796	42,858	16,420	9,562	2,075	5,244	910	1041 (-954, 4,491)
Cost-per-DALY averted relative to no further VMMC									
5 years	15,612	114,719	10,657	202,375	33,734	98,337	48,829	220,319	DALYs not averted (1,034, DALYs not averted)
20 years	180	1,522	265	47	845	4,480	1,339	3,585	1,245 (Cost saving ($0·5/yr, DALYs not averted)
50 years	VMMC dominates †	VMMC dominates	VMMC dominates	VMMC dominates	VMMC dominates	VMMC dominates	VMMC dominates	616	60 (VMMC dominates, DALYs not averted)[2]
Difference in net DALYs comparing continued VMMC vs. no VMMC assuming CET of US$500 per DALY averted (mean per year over time horizon)									
5 years	67,056 additional	63,186 additional	114,075 additional	50,600 additional	67,039 additional	25,506 additional	51,615 additional	19,801 additional	12,096 additional (20,670 additional, 1,443 additional)
20 years	17,160 additional	7,971 additional	6,370 averted	8,283 averted	5,394 additional	4,548 additional	6,517 additional	4,083 additional	1,284 additional (5,039 additional, 4,184 averted)
50 years	64,020 averted	11,552 averted	72,379 averted	42,956 averted	11,045 averted	2,259 averted	6,084 averted	210 additional	1,267 averted (1,821 additional, 7,792 averted)

*2021 US $

[1]Optima HIV costs include only VMMC and ART costs.

† Continuation of VMMC dominates no VMMC, offering both health benefits (infections, DALYs averted) and reduced costs.

[2]Assuming maximum difference in costs and one infection averted for setting scenarios in which infections are not averted.

Table A3: Using HIV Synthesis, associations between baseline outputs and cost-effectiveness over 50-year time horizon

Based on 200 setting scenarios

Output	Univariate odds ratio	p-value	Multivariate[1] odds ratio	p-value
Incidence in 2021/ 100 person years<br>Per 1 unit increase	11·53 (2·56, 51·82)	0·001	12·52 (2·58, 60·82)	0·002
% of all HIV positive people with VL< 1000 copies/mL in 2021<br>Per 1 unit increase	0·99 (0·95, 1·03)	0·62	1·00 (0·97, 1·04)	0·84
Proportion circumcised by 2021<br>Per 1 unit increase	1·01 (0·99, 1·03)	0·17	1·01 (1·00, 1·03)	0·13

Table A4: Costs (US$) and Disability Adjusted Life Years (DALYs) stratified by model, region, and time horizon (costs and DALYs discounted at 3% per year)

VMMC cost = US$60

**Setting**	**South Africa**				**Malawi**		**Zimbabwe**		**Setting scenarios**
**Model**	**Goals-ASM**	**Optima HIV**	**EMOD**	**Thembisa**	**Goals-ASM**	**Optima HIV**	**Goals-ASM**	**Optima HIV**	**HIV-Synthesis**
Mean annual total programme costs (not just VMMC) per 10 million[1] people over time horizon for adults age 15–64 (US$, millions per year) (No further VMMC/ continuation of VMMC for 5 years)									
5 years	398/404	268/273	286/292	346/ 350	199/221	139/147	255/277	249/258	142 (50, 326) / 147 (53, 330)
20 years	254/254	230/231	249/248	265/ 265	159/163	116/118	193/197	221/223	113 (38, 272) / 114 (38, 273)
50 years	154/151	176/175	165/162	150/ 148	113/111	85/85	125/123	180/180	80 (24, 205) / 80 (25, 205)
Difference in total costs per 10 million comparing continuation of VMMC for 5 years to no further VMMC (USD millions per year)									
5 years	+6	+5·1	+6·0	+3·8	+21·9	+7·8	+21·5	+8·4	+4·1 (+0·5, +6·9)
20 years	+0·6	+0·8	-1·0	-0·4	+6·4	+1·4	+3·7	+2·2	0·5 (-0·6, 1·6)
50 years	-2·5	-1·1	-3·0	-2·5	-1·8	-0·7	-1·6	+0·3	-0·4 (-2·3, 0·6)
Cost per infection averted (discounted difference in costs/(discounted infections averted)									
5 years	10,382	18,096	5121	9336	21,974	13,483	26,672	34,440	6391 (670, Infections not averted)
20 years	320	967	Cost saving ($1·0m/yr)	Cost saving ($0·4m/yr)	1,659	1,107	1,983	3,648	639 (VMMC dominates, Infections not averted)
50 years	VMMC dominates	VMMC dominates	VMMC dominates	VMMC dominates	VMMC dominates	VMMC dominates	VMMC dominates	643	VMMC dominates (VMMC dominates, Infections not averted)
Cost-per-DALY averted relative to no further VMMC									

Setting<br><br>Model	South Africa<br><br>Goals-ASM	 <br>Optima HIV	 <br>EMOD	 <br>Thembisa	Malawi<br><br>Goals-ASM	 <br>Optima HIV	Zimbabwe<br><br>Goals-ASM	 <br>Optima HIV	Setting scenarios<br><br>HIV-Synthesis
5 years	10,372	76,023	70673	134684	22,443	65,484	32,487	149,435	DALYs not averted (712, DALYs not averted)
20 years	83	802	VMMC dominates	VMMC dominates	481	2,492	777	2,259	732 (VMMC dominates, DALYs not averted)
50 years	VMMC dominates †	VMMC dominates	VMMC dominates	VMMC dominates	VMMC dominates	VMMC dominates	VMMC dominates	208	VMMC dominates (VMMC dominates, DALYs not averted)
Difference in net DALYs comparing continued VMMC vs. no VMMC assuming CET of US$500 per DALY averted (mean per year over time horizon)									
5 years	43,806 additional	41,991 additional	75,195 additional	33,633 additional	44,263 additional	16,942 additional	34,162 additional	13,181 additional	8119 additional (15078 additional, 786 additional)
20 years	22,973 averted	2,351 additional	17,253 averted	12,525 averted	301 averted	2,276 additional	2,154 additional	2,327 additional	268 additional (3933 additional, 4881 averted)
50 years	66,345 averted	13,917 averted	76,647 averted	44,652 averted	13,323 averted	3,218 averted	7,830 averted	531 averted	1750 averted (1340 additional, 8335 averted)

*2021 US $

[1]Optima HIV costs include only VMMC and ART costs.

† Continuation of VMMC dominates no VMMC, offering both health benefits (infections, DALYs averted) and reduced costs.

[2]Assuming maximum difference in costs and one infection averted for setting scenarios in which infections are not averted.

Table A5: Costs (US$) and Disability Adjusted Life Years (DALYs) stratified by model, region, and time horizon (costs and DALYs discounted at 3% per year)

VMMC cost = US$120

Setting	South Africa				Malawi		Zimbabwe		Setting scenarios
Model	Goals-ASM	Optima HIV	EMOD	Thembisa	Goals-ASM	Optima HIV	Goals-ASM	Optima HIV	HIV-Synthesis
Mean annual total programme costs (not just VMMC) per 10 million[1] people over time horizon for adults age 15–64 (US$, millions per year) (No further VMMC/ continuation of VMMC for 5 years)									
5 years	398/410	268/278	286/299	346/ 353	199/243	140/155	255/298	250/267	142 (50, 326) / 151 (55, 333)
20 years	254/256	230/232	249/251	265/ 266	159/168	116/120	193/202	221/226	113 (38, 272) / 115 (39, 274)
50 years	154/152	176/176	165/163	150/ 148	113/114	85/86	125/125	180/182	80 (24, 205) / 80 (25, 205)
Difference in total costs per 10 million comparing continuation of VMMC for 5 years to no further VMMC (USD millions per year)									
5 years	+12	+10·3	+13	+7·5	+43·9	+15·7	+43·1	+16·9	8·0 (1·0, 12·9)
20 years	+2·1	+2·3	+2·0	+0·5	+9·1	+3·6	+9·2	+4·8	1·5 (0·1, 2·9)
50 years	-1·9	-0·4	-2·0	-2·2	+0·4	+0·3	+0·6	+1·6	0·0 (-2·0, 1·1)
Cost per infection averted (discounted difference in costs/(discounted infections averted)									
5 years	20,872	36,236	10,381	18,720	44,085	27,012	53,505	69,144	12066 (1279, Infections not averted)
20 years	1,139	2,705	507	309	4,175	2,873	4,849	7,939	1807 (VMMC dominates, Infections not averted)
50 years	VMMC dominates †	VMMC dominates	VMMC dominates	VMMC dominates	210	419	325	3,163	68 (VMMC dominates, Infections not averted)
Cost-per-DALY averted relative to no further VMMC									

Setting	South Africa				Malawi		Zimbabwe		Setting scenarios
**Model**	**Goals-ASM**	**Optima HIV**	**EMOD**	**Thembisa**	**Goals-ASM**	**Optima HIV**	**Goals-ASM**	**Optima HIV**	**HIV-Synthesis**
5 years	20,852	152,634	143,241	270,065	45,025	131,190	65,171	296,622	DALYs not averted (1359, DALYs not averted)
20 years	294	2,243	667	279	1,210	6,468	1,901	4,912	1924 (55, DALYs not averted)
50 years	VMMC dominates	VMMC dominates	VMMC dominates	VMMC dominates	42	187	86	1,023	183 (VMMC dominates, DALYs not averted)
Difference in net DALYs comparing continued VMMC vs. no VMMC assuming CET of US$500 per DALY averted (mean per year over time horizon)									
5 years	90,306 additional	84,379 additional	152,956 additional	67,566 additional	69,068 additional	34,071 additional	68,682 additional	26,421 additional	16184 additional (26491 additional, 2062 additional)
20 years	11,348 averted	13,591 additional	4,513 additional	4,041 averted	10,880 additional	6,820 additional	7,800 additional	5,839 additional	2,248 additional (6,162 additional, 3,417 averted)
50 years	61,695 averted	9,185 averted	68,112 averted	41,259 averted	4,339 averted	1,299 averted	7,754 averted	952 additional	827 averted (2,355 additional, 7,378 averted)

*2021 US $

[1]Optima HIV costs include only VMMC and ART costs.

[†] Continuation of VMMC dominates no VMMC, offering both health benefits (infections, DALYs averted) and reduced costs.

[2]Assuming maximum difference in costs and one infection averted for setting scenarios in which infections are not averted.

Table A6: Modelled outputs in 2041 assuming continuation of VMMC and 20% lower ART coverage in 2041

**Setting**	**South Africa**				**Malawi**		**Zimbabwe**		**Setting scenarios**
**Model**	**Goals-ASM**	**Optima HIV Mean (90% range)**	**EMOD Mean (90% range)**	**Thembisa Mean (90% range)**	**Goals-ASM**	**Optima HIV Mean (90% range)**	**Goals-ASM**	**Optima HIV Mean (90% range)**	**HIV-Synthesis Median (90% range)**
HIV prevalence (%) (age 15–49)	6·4	10·0 (7·8, 15·2)	10·8 (10·2, 11·6)	8·5 (8·0–9·0)	2·8	5·6 (5·0, 8·2)	3·6	7·0 (6·3, 10·6)	3·0 (0·3, 12·6)
HIV incidence/100 person years (age 15–49)	0·36	0·34 (0·25, 0·45)	0·68 (0·63, 0·75)	0·28 (0·25, 0·32)	0·14	0·40 (0·24, 0·49)	0·17	0·26 (0·09, 0·35)	0·28 (0·03, 1·31)
% of all HIV positive people with VL < 1000 copies/mL (age 15–64)	51·7	43·6 (43·0, 43·6)	61·1 (60·8, 61·4)	58·2 (57·8, 58·5)	63	49·5 (49·1, 50·4)	59·6	56·2 (55·5 ,65·9)	53·0 (35·1, 68·0)
% of all HIV positive people on ART (age 15–64)	59·5	52·5 (47·5, 57·3)		61·4 (61·0, 61·6)	69·6	63·0 (58·5, 67·2)	70·0	70·7 (67·6, 74·5)	55·9 (37·2, 74·5)
% males ever circumcised[2]									
**15–49**	**63·3**	**50·7**	**61·4 (61·2, 61·5)**	**68·4**	**48·0**	**30·2**	**42·0**	**32·6**	**43·4 (14·0, 93·2)**
15–19	25·7	39·7	8·1 (7·9, 8·2)	20·2	16·4	15·1	7·3	11·6	10·0 (4·7, 90·2)
20–24	38·7	39·7	23·0 (22·7, 23·3)	31·4	18·8	18·7	9·0	17·5	10·0 (4·4, 90·3)
25–29	26·4	54·0	40·7 (40·4, 41·1)	80·6	20·3	25·3	10·3	38·2	13·0 (5·3, 91·3)
30–34	90·0	54·0	95·3 (95·1, 95·4)	96·0	90·0	34·7	90·0	38·2	72·3 (16·4, 95·0)
35–39	90·0	54·0	98·5 (98·4, 98·6)	94·8	90·0	44·1	90·0	47·8	82·1 (21·9, 97·5)
40–49	90·0	54·0	97·4 (97·2, 97·5)	89·5	90·0	50·1	90·0	47·8	80·3 (20·6, 96·9)
45–49	78·6	54·0	76·8 (76·5, 77·2)	78·0	51·1	52·5	40·2	47·8	73·0 (20·8, 95·3)

Table A7: **Costs and Disability Adjusted Life Years (DALYs) over 50-year time horizon, stratified by model and region assuming 20% lower ART coverage in 2040 (costs and DALYs discounted at 3% per year). VMMC cost assumed to be US$90**

**Setting**	**South Africa**				**Malawi**		**Zimbabwe**		**Setting scenarios**
**Model**	**Goals-ASM**	**Optima HIV**	**EMOD**	**Thembisa**	**Goals-ASM**	**Optima HIV**	**Goals-ASM**	**Optima HIV**	**HIV-Synthesis**
Mean annual total programme costs (not just VMMC) per 10 million[1] people over time horizon for adults age 15–64 (US$, millions per year) (No further VMMC/ continuation of VMMC for 5 years)									
50 years	131/129	169/168	158 / 155	144 / 141	111/108	87/86	117/115	160/161	58 (17, 180) / 58 (18, 180)
Difference in total costs comparing continuation of VMMC for 5 years to no further VMMC (USD millions per year)									
50 years	-2·2	-1·1	-3·0	-2·8	-2·9	-1·1	-2·5	+0·7	0·2 (-2·5, 2·1)
Total ART costs per 10 million over time horizon for adults age 15–64 (USD, millions per year) (No further VMMC/ continuation of VMMC)									
50 years	120/117	169/167	114/111	138/135	83/77	87/85	103/97	160/159	37 (5, 139)/ 36 (5, 139)
Difference in total ART costs comparing continuation of VMMC to no further VMMC (USD millions per year)									
50 years	-3·1	-2·1	-3·0	3·3	-6·2	-2·6	-5·7	-1·2	-0·3 (-3·0, 1·4)
Cost per infection averted (discounted difference in costs/(discounted infections averted)									
50 years	VMMC dominates	VMMC dominates	VMMC dominates	VMMC dominates	VMMC dominates	VMMC dominates	VMMC dominates	943	391 (VMMC dominates, Infections not averted)
Difference in DALYs comparing continued VMMC vs. no VMMC (mean per year over time horizon, 3% discount rate)									

Setting	South Africa				Malawi		Zimbabwe		Setting scenarios
**Model**	**Goals-ASM**	**Optima HIV**	**EMOD**	**Thembisa**	**Goals-ASM**	**Optima HIV**	**Goals-ASM**	**Optima HIV**	**HIV-Synthesis**
	83,962	10,247	60,395	21,892	25,909	2,551	15,255	1,479	7742 (-18272, 44973)
Cost-per-DALY averted relative to no further VMMC									
50 years	VMMC dominates	VMMC dominates	VMMC dominates	VMMC dominates	VMMC dominates	VMMC dominates	VMMC dominates	305	35 (VMMC dominates, DALYs not averted)
Difference in net DALYs comparing continued VMMC vs. no VMMC assuming CET of US$500 per DALY averted (mean per year over time horizon)									
50 years	100,859 averted	16,784 averted	90,061 averted	53,397 averted	31,931 averted	4,312 averted	19,251 averted	576 averted	7169 averted 22210 additional, 48292 averted)

*2021 US $

[1]Optima HIV costs include only VMMC and ART costs.

[†] Continuation of VMMC dominates no VMMC, offering both health benefits (infections, DALYs averted) and reduced costs.

[2]Assuming maximum difference in costs and one infection averted for setting scenarios in which infections are not averted.

Table A8: Same as primary analysis but Costs and Disability Adjusted Life Years (DALYs) over 50-year time horizon, stratified by model and region assuming costs and DALYs discounted at 5% per year, VMMC cost assumed to be US$90

Setting	South Africa				Malawi		Zimbabwe		Setting scenarios
Model	Goals-ASM	Optima HIV	EMOD	Thembisa	Goals-ASM	Optima HIV	Goals-ASM	Optima HIV	HIV-Synthesis
Mean annual total programme costs (not just VMMC) per 10 million[1] people over time horizon for adults age 15–64 (US$, millions per year) (No further VMMC/ continuation of VMMC for 5 years)									
50 years	113/112	193/193	118/117	111 / 110	78/79	95/96	89/90	194/196	80 (35, 216) / 79 (35, 214)
Difference in total costs comparing continuation of VMMC for 5 years to no further VMMC (USD millions per year)									
50 years	-0·7	+0·2	-1·0	-1·1	+1·0	+0·5	+1·0	+2·0	-0·2 (-1·7, 0·8)
Cost per infection averted (discounted difference in costs/(discounted infections averted)									
50 years	Cost saving ($0·7m/yr)	134	Cost saving ($1·0m/yr)	Cost saving ($1·1m/yr)	816	830	960	3,821	Cost saving ($0·2m/yr) (Cost saving ($1·7m/yr), 1400000)
Difference in DALYs comparing continued VMMC vs. no VMMC (mean per year over time horizon, 3% discount rate)									
50 years	21,527	3,670	21,572	7,742	4,577	1,075	2,544	514	2511 (-1242, 9553)
Cost-per-DALY averted relative to no further VMMC									
50 years	VMMC dominates	65	VMMC dominates	VMMC dominates	217	474	331	1,447	VMMC dominates (VMMC dominates, DALYs not averted)
Difference in net DALYs comparing continued VMMC vs. no VMMC assuming CET of US$500 per DALY averted (mean per year over time horizon)									
50 years	27,064	3,189	33,982	20,485	2,593	55	858 averted	973 additional	2657 averted (948 additional,

Setting	South Africa				Malawi		Zimbabwe		Setting scenarios
Model	Goals-ASM	Optima HIV	EMOD	Thembisa	Goals-ASM	Optima HIV	Goals-ASM	Optima HIV	HIV-Synthesis
	averted	averted	averted	averted	averted	averted			11424 averted)

*2021 US $

[1]Optima HIV costs include only VMMC and ART costs.

[†] Continuation of VMMC dominates no VMMC, offering both health benefits (infections, DALYs averted) and reduced costs.

[2]Assuming maximum difference in costs and one infection averted for setting scenarios in which infections are not averted.

Table A9: Key outputs from other African countries modelled by Goals-ASM

	**Botswana**	**Eswatini**	**Lesotho**	**Mozambique**	**Namibia**	**Rwanda**	**Uganda**	**Zambia**
HIV prevalence 2021 (15–49)	16·5%	23·3%	21·5%	11·5%	10·6%	2·1%	5·1%	9·5%
HIV incidence 2021 15–49)	0·16	0·22	0·86	0·60	0·23	0·01	0·15	0·17
% males ever circumcised 2021 (15–49)	43·0%	35·9%	38·7%	72·2%	47·1%	40·3%	50·2%	33·8%
Cost effectiveness analyses over 50 year time horizon								
Difference in total costs comparing continuation of VMMC for 5 years to no further VMMC (USD millions per year)	1·3	1	-3·1	-2	-0·2	2·6	-0·4	0·6
Difference in DALYs comparing no VMMC with continued VMMC (DALYs for no VMMC – DALYS for continued VMMC; mean per year over time horizon, 3% discount rate)	24	29	264	2295	75	15	1115	764
Cost-per-DALY averted for continued VMMC	932	487	VMMC dominates †	VMMC dominates	VMMC dominates	9515	VMMC dominates	92

relative to no further VMMC								

[†] Continuation of VMMC dominates no VMMC, offering both health benefits (infections, DALYs averted) and reduced costs.

Figure A1: **Zimbabwe – future incidence prediction modelled by Goals-ASM and Optima HIV**

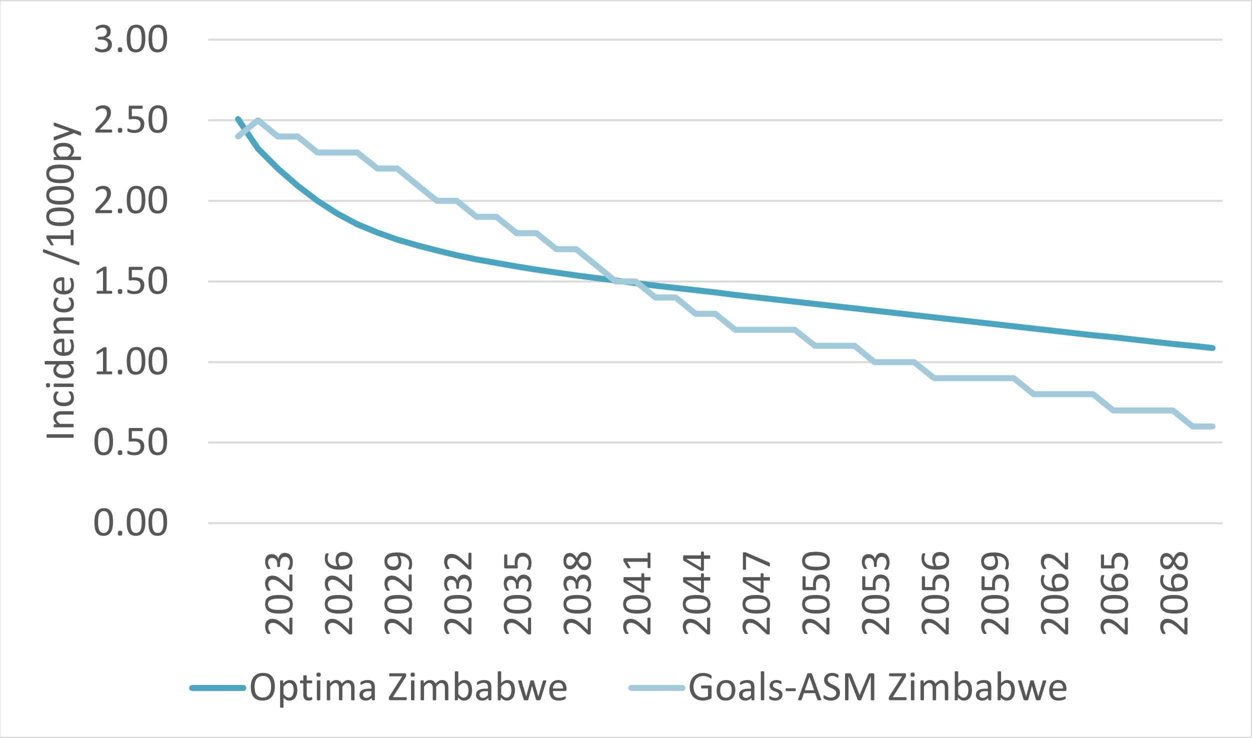

Supplement: Supplementary appendix [file mmc1.pdf]
